# miR-1279, miR-548j, miR-548m, and miR-548d-5p Binding Sites in CDSs of Paralogous and Orthologous *PTPN12*, *MSH6*, and *ZEB1* Genes

**DOI:** 10.1155/2013/902467

**Published:** 2013-07-16

**Authors:** Anatoliy T. Ivashchenko, Assel S. Issabekova, Olga A. Berillo

**Affiliations:** National Nanotechnology Laboratory, Al-Farabi Kazakh National University, Almaty 050038, Kazakhstan

## Abstract

Only *PTPN12, MSH6*, and *ZEB1* have significant miR-1279 binding sites among paralogous genes of human tyrosine phosphatase family, DNA mismatch repair family, and zinc finger family, respectively. All miRNA binding sites are located within CDSs of studied mRNAs. Nucleotide sequences of hsa-miR-1279 binding sites with mRNAs of human *PTPN12, MSH6*, and *ZEB1* genes encode TKEQYE, EGSSDE, and GEKPYE oligopeptides, respectively. The conservation of miRNA binding sites encoding oligopeptides has been revealed. MRNAs of many paralogs of zinc finger gene family have from 1 to 12 binding sites coding the same GEKPYE hexapeptide. MRNAs of *PTPN12, MSH6*, and *ZEB1* orthologous genes from different animal species have binding sites for hsa-miR-1279 which consist of homologous oligonucleotides encoding similar human oligopeptides TKEQYE, EGSSDE, and GEKPYE. MiR-548j, miR-548m, and miR-548d-5p have homologous binding sites in the mRNA of *PTPN12* orthologous genes which encode PRTRSC, TEATDI, and STASAT oligopeptides, respectively. All regions of miRNA are important for binding with the mRNA.

## 1. Introduction

Posttranscriptionally miRNAs regulate the expression of genes involved in organism development [1], metabolism [2], cell cycle, apoptosis [3], carcinogenesis [4], protein-protein interactions [5], and so forth. The specific functions of most identified miRNAs remain unknown, although some targets have been established. Computational algorithms have been developed to predict miRNA targets [6]. For animal miRNAs, target prediction is an issue because miRNAs can bind to target genes without perfect complementarity [7]. Only some predicted miRNA binding sites had been experimentally verified [8]. 

Many pieces of software initially used complementarity to identify potential binding sites. Subsequent filtering steps were based on thermodynamics, binding site structures, and site conservation across species [9]. The false-positive rate of predicting miRNA targets has resulted in the development of several algorithms: miRanda (http://www.microrna.org/microrna/home.do) [10], TargetScan (http://www.targetscan.org/) [11], and PicTar (http://pictar.mdc-berlin.de/) [12]. Other algorithms apply thermodynamics as the initial requirement for selection of interaction sites between miRNAs and their targets. DIANA-microT (http://diana.cslab.ece.ntua.gr/) [13] and RNAhybrid (http://bibiserv.techfak.uni-bielefeld.de/rnahybrid/) [14] belong to this type of software. We have used a local RNAHybrid software to identify miRNA target sites and have attempted to explain the benefits of this software [15]. The software allows the determination of the most energetically favourable interactions of small RNAs to mRNAs in all regions: 5′untranslated regions (5′UTR), coding domain sequences (CDS), and 3′untranslated regions (3′UTR). The majority of on-line pieces of software predict miRNA target sites only in the 3′UTR, and most of the characterised miRNA binding sites occur in this region [16]. Experimental investigations revealed the occurrence of 5′UTR and CDS miRNAs binding sites [16–18]. According to another study, human mRNAs contain many strong sites with 13 or more base pairings without bulges in the middle [19].

Previously, we had revealed miRNA binding sites in 54 mRNAs of oncogenes including *PTPN12, MSH6, *and* ZEB1 *that had sites in CDSs with a high Δ*G*/Δ*G*_*m*_ ratio [19]. These genes are involved in the development of breast cancer [20], colorectal cancer [21], lymphoma [22], and esophageal cancer [23]. The *PTPN12* gene encodes a protein from tyrosine phosphatase family. It participates in different cellular processes, including cell growth, the mitotic cycle, and oncogenic transformation [24]. *MSH6* is a DNA mismatch repair protein that participates in the recognition of mismatched nucleotides prior to their repair [25]. Mutations in this gene have been associated with hereditary nonpolyposis colon cancer and endometrial cancer [26]. The *ZEB1* gene encodes a protein which belongs to a large family of zinc finger transcriptional factors [27]. Identification of the role of miRNA in the regulation of the gene and the gene set expression is an important problem. Thus, we have examined the presence of miR-1279 target sites in paralogous genes for *PTPN12, MSH6, *and* ZEB1*. 

MiRNA sites are localized within the 5′UTR, CDS, and 3′UTR of mRNA [28]. Indeed, about half of all miRNA binding sites are located in the protein-coding region [29] suggesting that studies should pay much attention to these sites. The nucleotide placement of these binding sites coding the amino acid sequence may raise important questions about binding sites localized in the CDSs.

To reduce the number of false positives, target sites may be validated by conservation of sites across species. To verify predicted sites by RNAHybrid, we have examined conservation of these sites across orthologous genes of different organisms. Significant sites in human genes have been investigated in *PTPN12, MSH6, *and* ZEB1* orthologues. Investigating these problems is necessary to establish the role of miRNA in the regulation of single gene and gene sets expressions. 

In present work we analysed the conservation of miR-1279, miR-548 m, miR-548j and miR-548d-5p binding sites inorthologues and paralogues of the *PTPN12*, *MSH6* and *ZEB1* genes to verify these interactions.

## 2. Materials and Methods

Investigation objects were mRNAs of *PTPN12*, *MSH6,* and *ZEB1* human genes, their orthologs, and paralogs (Supplementary Tables S1, S2 in Supplementary Material available online at http://dx.doi.org/10.1155/2013/902467) in *Anolis carolinensis *(Aca),* Ailuropoda melanoleuca *(Ame)*, Bos taurus *(Bta)*, Cricetulus griseus *(Cgr)*, Cavia porcellus *(Cpo)*, Callithrix jacchus *(Cja)*, Canis lupus familiaris *(Clf)*, Danio rerio *(Dre)*, Equus caballus *(Eca)*, Gallus gallus *(Gga)*, Homo sapiens (Hsa), Loxodonta africana *(Laf)*, Macaca mulatta *(Mml)*, Monodelphis domestica *(Mdo)*, Meleagris gallopavo *(Mga)*, Mus musculus *(Mmu)*, Nomascus leucogenys *(Nle)*, Ornithorhynchus anatinus *(Oan)*, Oryctolagus cuniculus *(Ocu)*, Oreochromis niloticus *(Oni)*, Otolemur garnettii *(Oga)*, Pan paniscus *(Ppa)*, Papio anubis *(Pan)*, Pan troglodytes *(Ptr)*, Pongo abelii *(Pab)*, Rattus norvegicus *(Rno)*, Saimiri boliviensis *(Sbo)*, Sarcophilus harrisii *(Sha)*, Sus scrofa *(Ssc)*, Taeniopygia guttata *(Tgu)*, Xenopus laevis *(Xla), and* Xenopus tropicalis *(Xtr). Nucleotide and amino acid sequences were accessed from GenBank (http://www.ncbi.nlm.nih.gov/). Nucleotide sequence of hsa-miR-1279, hsa-miR-548 m, and hsa-miR-548j were received from miRBase (http://www.mirbase.org/).

Free energy (Δ*G*), position of potential binding sites, and interaction schemes were calculated by RNAhybrid 2.1 software (http://bibiserv.techfak.uni-bielefeld.de/rnahybrid/). The E-RNAhybrid software (http://sites.google.com/site/malaheenee/software/) was used to compute the ratio of Δ*G*/Δ*G*_*m*_ and *P*value. Δ*G*/Δ*G*_*m*_ value equalled 75% and more was used as comparative criterion of miRNA and mRNA interaction force. Δ*G*/Δ*G*_*m*_ value shows degree of complementarity of each miRNA site in target gene. Δ*G* value and its standard deviation were used to determine significance level of miRNA binding sites with mRNA. Interaction energies (Δ*G*_*m*_) of hsa-miR-1279, hsa-miR-548 m, hsa-miR-548 j, and hsa-miR-548 d-5 p with their perfectly complementary sequence were calculated and equaled −118 kJ/moL, −139 kJ/moL, −161 kJ/moL, and −148 kJ/moL, consequently. At first, miRNA binding sites were found for human mRNAs, and then they were revealed in homologous and paralogous genes of studied animals. Diagrams of nucleotide and protein variability were produced by WebLogo software (http://weblogo.threeplusone.com/). 

## 3. Results

### 3.1. Features of hsa-miR-1279 Binding Sites in mRNAs of Paralogous and Orthologous *PTPN12* Genes

To investigate the role of miR-1279 in the regulation of tyrosine phosphatase family genes, we attempted to elucidate target binding sites among them. We identified the *PTPN12* gene as a reliable target of hsa-miR-1279 with binding energy of −102.1 kJ/moL and a Δ*G*/Δ*G*_*m*_ value of 86.5%. hsa-miR-1279 consists of 17 nucleotides; 15 of these nucleotides are perfectly complementary to the binding site of human *PTPN12* mRNA. We considered miRNA binding sites with Δ*G*/Δ*G*_*m*_ values greater than 75%. All sites with lower values were determined to be weak sites and were not considered. The hybridization energy of miR-1279 with the mRNA of tyrosine phosphatase family genes, except that of *PTPN12*, varied from −67.7 kJ/moL to −89.5 kJ/moL. Corresponding Δ*G*/Δ*G*_*m*_ values ranged from 57.4% to 75.0%. Thus, only *PTPN12* was considered. The nucleotide sequence of the binding site in PTPN12 encoded the TKEQYE hexapeptide, which was not a conserved amino acid sequence in any other tyrosine phosphatase family. Consequently, miR-1279 may efficiently regulate only *PTPN12* gene expression.

**Figure 1:**
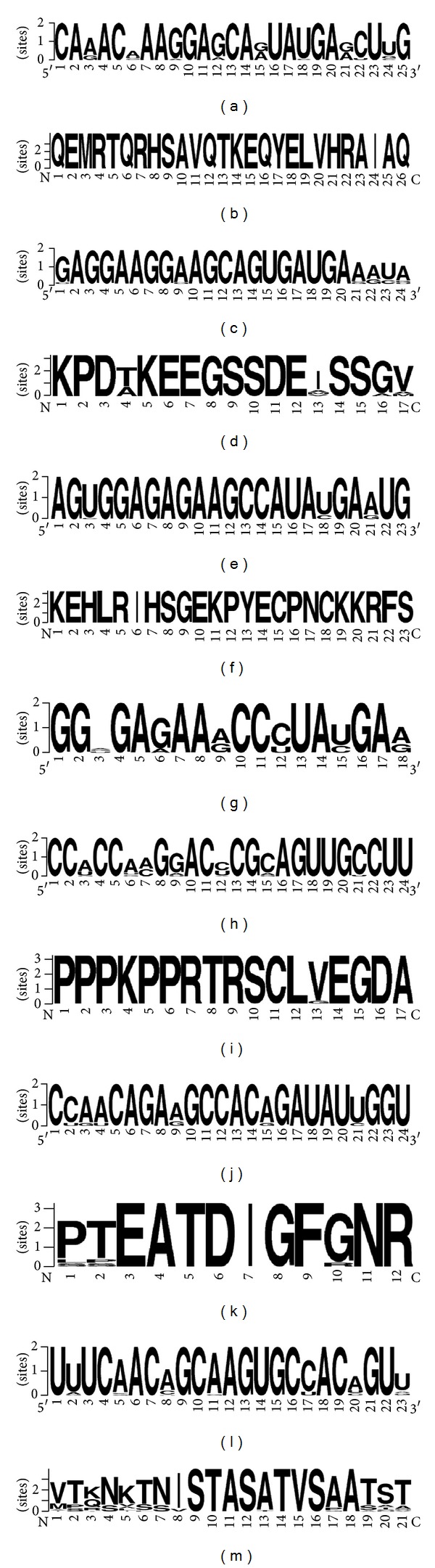
Fragments of nucleotide and amino acid sequences, where miRNA binding sites are located. The variability of nucleotides in miR-1279 binding sites in mRNA of *PTPN12* (a), *MSH6* (c), and *ZEB1* (e) orthologous genes and the variability of amino acids of PTPN12 (b), MSH6 (d), and ZEB1 (f) orthologous proteins; miR-1279 binding site encode TKEQYE (b), EGSSDE (d), and GEKPYE (f) oligopeptides; (g), the variability of nucleotides in miR-1279 binding sites of zinc-finger transcriptional factors family; (h), (j) and (l)—the variability of nucleotides in 548j, 548 m, and miR-548d-5p binding sites in mRNA of *PTPN12*, respectively; (i), (k), and (m), the variability of amino acids of PTPN12 orthologous proteins in regions with PRTRSC, EATDI, and STASAT oligopeptides, respectively.

Furthermore, we investigated the conservation of the miR-1279 binding site in orthologous genes. The human *PTPN12* gene has 23 orthologues in different animals. For determination of the conservation of the relevant site, we verified the nucleotide sequence of the binding site. The nucleotide sequences of these sites in the mRNA of the investigated genes are presented in Table 1. Sequences of the mRNA *PTPN12* binding sites in all animals were homologous, and nucleotides in first and second positions of the codons were conserved (Figure 1(a)). ACGAAGGAGCAGUAUGAA oligonucleotide of the interaction site in the CDS encoded the TKEQYE hexapeptide, which was identical in all orthologous proteins of the studied species (Table 1). Replacements in the third position of the codon in described oligonucleotide sequences did not change the amino acid content. This oligopeptide was located from positions13 to 18 in the conserved amino acid sequences of tyrosine phosphatase orthologues (Figure 1(b)). These data indicate that the main function of this site is gene regulation by miR-1279. All miR-1279 interaction sites in *PTPN12* orthologues corresponded to an open frame. Replacements of one or several nucleotides in the third position of the codon led to decreased binding energies, but did not change the location of the miR-1279 binding site. Interactions between hsa-miR-1279 and the mRNA of *PTPN12* orthologues showed significance levels of Δ*G*/Δ*G*_*m*_ values ranging from 71.3% to 87.9% (Table 1). MiRNA binding sites were identical in 11 species from mammals to amphibians.

The binding energy of orthologous mRNA with orthologous miRNA may vary if the sequences of orthologous miRNAs in animals differ from that of hsa-miR-1279. It is possible that the interactions of hsa-miR-1279 with the mRNA of *A*. *carolinensis*, *C*. *jacchus*, *D*. *rerio*, *M*. *domestica*, and *X*. *laevis* tyrosine phosphatase orthologues are less effective. These data confirm that this binding site exists from the early stages of evolution and did not change for tens to hundreds of millions of years. 

### 3.2. Features of hsa-miR-1279 Binding Sites in mRNAs of Paralogous and Orthologs *MSH6* Genes

The human genome encodes several DNA mismatch repair proteins: *MSH2*, *MSH3*, *MSH4*, *MSH5*, *MSH6*, *MLH1*, and *MLH3*. We did not find conserved strong miR-1279 binding sites in the mRNAs of these genes, with the exception of the *MSH6* gene, among all considered DNA mismatch repair proteins. Nonconserved miR-1279 interaction sites in these genes had Δ*G*/Δ*G*_*m*_ values ranging from 62.0% to 80.5%, and only the *MLH3* gene had a significant binding site (Δ*G*/Δ*G*_*m*_ value is 73%). These data confirmed the specific binding of miR-1279 with *MSH6* mRNA among genes of the DNA mismatch repair family.

The hsa-miR-1279 binding site in human *MSH6* mRNA had perfect complementary nucleotides with a single nucleotide bulge, including 4 G-U base pairs, in spite of its high Δ*G*/Δ*G*_*m*_ value (89.4%) and binding energy (−105.4 kJ/moL). The GAAGGAAGCAGUGAUGA oligonucleotide sequence in the binding site of *MSH6* mRNA (Figure 1(c)) encoded the EGSSDE hexapeptide in MSH6 protein (Table 2). We observed 34 orthologues for human *MSH6* and miR-1279 binding sites with high complementarily levels in 15 orthologues. Oligonucleotides of these sites and corresponding oligopeptides are shown in Table 2. MSH6 protein from 13 mammals and human contained completely homologous EGSSDE hexapeptides and the corresponding nucleotide sequences in mRNA binding sites exhibited high homology. There was nucleotide variability in the third position of the glutamic acid codons at the beginning of the EGSSDE hexapeptide, situated from positions 13 to 30 in the highly conserved protein region of MSH6 (Figure 1(d)). These data suggest the functional importance of the miR-1279 binding site in the regulation of *MSH6* gene expression. 

All binding sites in *MSH6* orthologous mRNA corresponded to an open reading frame. Replacement of one or several nucleotides in the third position of codons led to diminished hybridization energy but did not change the location of the hsa-miR-1279 binding site. Such replacements did not influence the amino acid composition properties of the protein. The Δ*G*/Δ*G*_*m*_ value of interaction sites varied from 87.6% to 89.4% (Table 2). 

### 3.3. Features of hsa-miR-1279 Binding Sites in mRNAs of Human ZNF Family and *ZEB1* Orthologous Genes

The *ZEB1* gene is a member of the large ZNF gene family of transcription factors. We searched for miR-1279 binding sites in the CDSs of several genes from this family. *ZNF552* and *ZNF790* mRNAs had miR-1279 sites, where the Δ*G*/Δ*G*_*m*_ values equalled 89.4% and 87.9%, respectively. Nucleotide sequences of the binding sites in these 2 genes shared high homology and encoded a GEKPYE oligopeptide. In fact, while many genes of this family had the GEKPYE oligopeptide, not all were targets for miR-1279. There were no significant binding sites for hsa-miR-1279 in *ZNF91*, *ZNF148*, *ZNF208*, *ZNF232*, *ZNF236*, *ZNF423*, *ZNF495B*, *ZNF509*, *ZNF521*, *ZNF729*, *ZNF776*, *ZNF768*, *ZNF853*, *ZNF857A*, and *ZNF865* genes within the CDS, and these genes did not encode the GEKPYE oligopeptide.

Proteins encoded by *ZEB2*, *ZNF8*, *ZNF70*, *ZNF84*, *ZNF140*, *ZNF454*, *ZNF461*, *ZNF475*, *ZNF529*, *ZNF576*, *ZNF594*, *ZNF713*, *ZNF751*, and *ZNF755* genes had this oligopeptide in structure, but the corresponding oligonucleotides were bound by hsa-miR-1279 with low hybridization energies. These oligonucleotides differed from the human *ZEB1* gene sequence by replacements in the third positions of several codons (Figure 1(g)). These genes have between 1 and 7 sites encoding the GEKPYE hexapeptide. We assume that if miR-1279 would be bound to these sequences, gene expression of these proteins would be inhibited. The expression of majority of the genes in the zinc-finger transcription factor family was not regulated by miR-1279.

The strongest binding site in genes of the zinc-finger transcription factor family was found in *ZEB1* mRNA. The binding energy of miR-1279 with *ZEB1* mRNA was 90% of the Δ*G*_*m*_, and 16 out of 17 nucleotides from the hsa-miR-1279 sequence exhibited total complementarity. Thus, it is suggested that they may interact in vivo. To verify the miR-1279 binding site in the *ZEB1* gene, we investigated the interactions between hsa-miR-1279 and mRNAs of *ZEB1* orthologues from 12 animal species. The human *ZEB1* gene has 16 identified orthologues, but only 12 of them retained this hsa-miR-1279 binding site and had conserved amino acid sequences in the binding region. The nucleotide sequence of the miR-1279 binding site (GGAGAGAAGCCAUAUGA) had high homology between these 12 orthologues (Figure 1(e)). The third nucleotide of these codons sequences encoded changes in tyrosine and glutamic acid, but these changes did not influence the encoding of the GEKPYE oligopeptide. This oligopeptide was localized in the conserved region of orthologous proteins (Figure 1(f)). Characteristics of the interactions between hsa-miR-1279 and *ZEB1* orthologue mRNAs of different animals are shown in Table 3. This binding site was well conserved among mammals and birds but was found in amphibians with less accuracy.

### 3.4. Features of hsa-miR-548j Binding Sites in mRNAs of *PTPN12* Orthologous Genes

*PTPN12* mRNA contained near perfect binding sites for several miRNAs (miR-1279, miR-548j, and miR-548 m). In the first part of this study, we validated miR-1279 binding site conservation across species. Furthermore, we considered the conservation of miR-548j interaction sites in *PTPN12* mRNA. MiR-548j has intronic origins (intron 9 in the *TPST2* gene). The nucleotide sequence of the miR-548j binding site in the CDS of *PTPN12* mRNA exhibited low variability and was present in all investigated organisms from mammals to fish (Figure 1(h)). Characteristics of these interactions are presented in Table 4. The Δ*G*_*m*_ value of miR-548j with a completely complementary sequence equalled −161 kJ/moL. The nucleotide sequence of the miR-548j binding site corresponded to the PRTRSC oligopeptide. This hexapeptide did not change across 16 species of mammals, reptiles, and birds but exhibited some changes in 4 species of fishes and amphibians (Figure 1(i)). Such conservation of revealed binding sites indicates the possibility of regulator role of miR-548j in *PTPN12* gene expression.

 The third miRNA that binds to *PTPN12* mRNA was miR-548 m. This binding site showed a lower level of conservation than miR-1279 and miR-548j binding sites. MiR-548 m interacted with the site encoding a conserved TEATDI hexapeptide in 7 species (Figure 1 (k)). A homologous oligopeptide from other animal species exhibited mismatches in 1 or 2 amino acids but had a similar position in a conserved region of the protein. The Δ*G*_*m*_ value of miR-548 m with a perfectly complementary sequence equalled −139.4 kJ/moL. Characteristics of miR-548 m binding sites are presented in Table 5 and showed a high level of conservation. In fact, the binding site only had perfect conservation across 4 investigated species (chimpanzee, gibbon, horse, and elephant). The Δ*G*/Δ*G*_*m*_ values vary from −75.1% to −80.8% (Table 5). Consequently, the effect of miR-548 m on *PTPN12* mRNA expression was less than those of miR-1279 and miR-548j.

### 3.5. Features of hsa-miR-548d-5p Binding Sites in mRNAs of *PTPN12* Orthologous Genes

MiR-548d-5p had another binding site in *PTPN12* mRNA. The binding energy of this site was lower than in the miR-548j and miR-548 m binding sites, but the nucleotide sequence in this site exhibited conservation in orthologous genes (Table 6). The amino acid sequences up- and downstream of the hexapeptide STASAT of PTPN12 were variable (Figure 1 (m)). The nucleotide sequences of the miR-548d-5p binding sites and *PTPN12* mRNA were homologous (Figure 1(l)); however, nucleotides in the third codon position were variable, and thus, these variable nucleotides did not cause changes in the amino acids coding of hexapeptide STASAT in some cases.

### 3.6. Nucleotide Interactions of hsa-miR-1279 Binding Sites with mRNAs of *PTPN12*, *MSH6*, and *ZEB1*

Next, we investigated the regions of the miRNA that are important for target gene regulation. Binding sites may be different according to the primary contribution of the specific region of miRNA to the hybridization energy. We observed 3 types of miRNA contributions: (1) the contribution of 5′-end dominated, (2) the contribution of the central region dominated, and (3) the contribution of 3′-end dominated (Table 7). A schematic representation of the nucleotide sequences of hsa-miR-1279 binding sites in the mRNA of *PTPN12*, *MSH6*, and *ZEB1* from different animals is shown in Table 7. The miR-1279 binding site in the *PTPN12* gene of 14 animal species was 5′-dominant and evolutionary conserved. *PTPN12* mRNAs from *A. melanoleuca*, *C. lupus*, *E. caballus*, and *O. niloticus* also had perfect complementarity in 5′-end of miR-1279 but had a reduced number of conserved nucleotides than the first gene group and had several mismatches in the target region. mRNAs of *MSH6* from 11 animal species had 3′-dominant miR-1279 sites and were highly homologous in all species. The 3′-end of miR-1279 was the primary contributor in these binding sites. MiR-548j was bound to the mRNA of the investigated species through the central region or the 3′-end in *O. niloticus* and *D. rerio* (Table 7). As shown in Table 7, miR-548 m and miR-548d-5p interacted predominantly with the 3′-end and, in several cases, the central region (*A. carolinensis*, *C. jacchus*, *Gallus gallus*, *M. gallopavo*, *O. niloticus,* and *P. abelii*). All described data confirmed that the nucleotide sequences of mRNA site may bind with all miRNA regions. 

## 4. Discussion

Previously, it was shown that 54 human mRNAs involved in oncogenesis contained strong miRNA binding sites within their CDSs that were predicted by RNAhybrid [19]. Interactions between mRNAs and miRNAs in binding sites were selected using Δ*G*/Δ*G*_*m*_ values, allowing us to identify miRNA interactions with near perfect complementarity. We examined the variation in sequences of binding sites and have been able to show good conservation of strong binding sites among the organisms we have investigated. 

It was predicted that miR-127 was 9 bound to mRNA of *PTPN12*, *MHS6*, and *ZEB1* in sites encoding 3 different oligopeptides (Tables 2, 4, and 6). miRNA binding sites may correspond to 3 open reading frames. Interactions between *PTPN12*, *MSH6*, and *ZEB1* mRNAs and miR-1279, as well as interactions between *PTPN12* mRNA and miR-548j, miR-548 m, and miR-548d-5p correspond to different open reading frames in their mRNA targets. The ability of miRNA binding sites to encode 1 oligopeptide suggests the stable influence of miRNA on mRNA during evolution. 

Similar conservation of nucleotide regions in CDSs of studied mRNAs and corresponding amino acid of proteins has been established for human *APC*, *BAD*, *EPHB2*, and *MMP2* genes (unpublished data). MiRNA binding sites in plant proteins encode regions of mRNAs in paralogous genes of the SPL family with high homology of oligonucleotides and corresponding hexapeptides (unpublished data). The nucleotide sequences adjacent to binding sites and to the corresponding amino acids have high variability (Figure 1). Highly homologous miRNA binding sites of orthologous genes and conservative oligopeptides of corresponding proteins have been established. 

The binding sites of several miRNAs are located in the 3′UTRs of some orthologous genes in the Drosophila genome and have highly homologous nucleotide sequences [30]. All nucleotides of miRNAs that participate in forming binding sites with mRNA in plants are highly conserved and not only in seeds [31]. These data prove the high conservatism of many miRNAs and show the importance of all nucleotide sequences of miRNAs with respect to binding to mRNAs. The presence of many target genes for a single miRNA suggests the functional connection of these genes. According to the data collected for the zinc-finger transcription factor family, the presence of oligopeptides that associate with the miRNA binding site in mRNA is not necessarily sufficient for miRNA site prediction. Changes in the third position of codons may cause weakening of the miRNA binding site or result in lack of binding ability. We have found this effect in many genes from the zinc-finger family. Mutations in the third position of codons can lead to loss of functional importance of miRNA binding. 

Target site conservation is one of the primary prediction validation methods. We have used this prediction to evaluate several factors. Investigation of 3 different gene families demonstrated that miR-1279 efficiently regulated only 1 gene from the tyrosine phosphatase family and DNA mismatch repair family. Furthermore, miR-1279 affected several mRNAs from paralogous genes of the zinc-finger transcription factor family.

Single miRNAs can also affect gene sets by conservation of the target site in orthologous genes. MiR-1279 has been shown to have a high probability of regulating *PTPN12*, *MHS6*, and *ZEB1*. Estimation of the degree of binding of miRNA with orthologous mRNAs has demonstrated that the degree of complementarity increases from fish and amphibians to mammals and is identical across primates. Similar trends have been identified in the *ZEB1* gene. Additionally, several miRNAs have been confirmed to affect a single gene. These data support that miR-1279, miR-548j, miR-548 m, and miR-548d-5p may potentially regulate *PTPN12* gene expression.

## 5. Conclusion

High conservation of binding sites in orthologous genes has proven the importance and antiquity of miRNA interactions with mRNAs of target genes. The expression of some paralogous genes can be regulated by a single miRNA. However, repression of the expression of entire gene families via 1 miRNA is unlikely. The expression of one gene can be regulated by several miRNAs, presupposing multiple methods of gene regulation that include dependence on the expression of intronic miRNAs. Establishment of multiple miRNA interactions with mRNAs is a complex problem, but it is necessary for definite regulation of gene expression through miRNAs. The power of miRNA linkage with mRNAs in CDSs can change at the expense of nucleotide replacement in the third position of the codons. This phenomenon has been clearly shown in miR-1279 binding sites in the mRNAs of human ZNF family genes.

## Supplementary Material

Accession numbers of studied orthologous genes are presented in Supplementary Table S1, S2.

## Figures and Tables

**Table 1:** Characteristics of miR-1279 binding sites in CDSs of *PTPN12* orthologous genes.

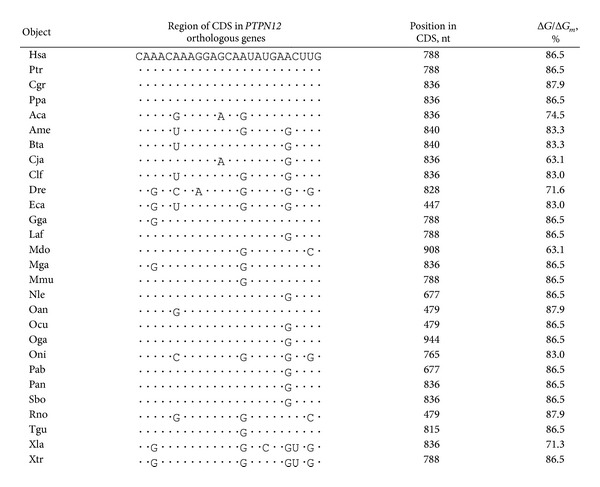

Note: the dots are identical nucleotides or amino acids in data presented in Tables 1–6.

**Table 2:** Characteristics of miR-1279 binding sites in CDSs of *MSH6* orthologous genes and the variability of amino acids of MSH6 orthologous proteins in regions with EGSSDE oligopeptide.

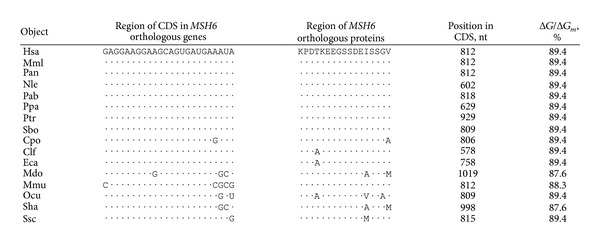

**Table 3:** Characteristics of miR-1279 binding sites in CDSs of *ZEB1*  orthologous genes.

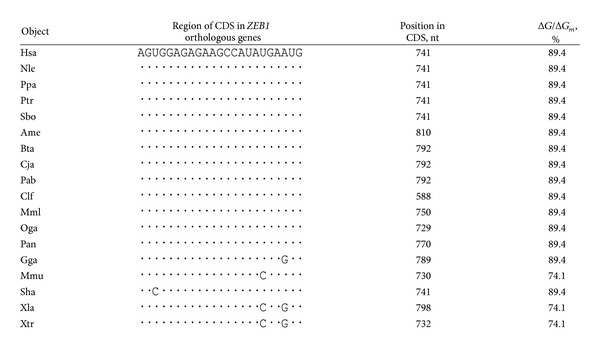

**Table 4:** Characteristics of miR-548j binding sites in CDSs of *PTPN12* orthologous genes and the variability of amino acids of PTPN12 orthologous proteins in regions with PRTRSC oligopeptide.

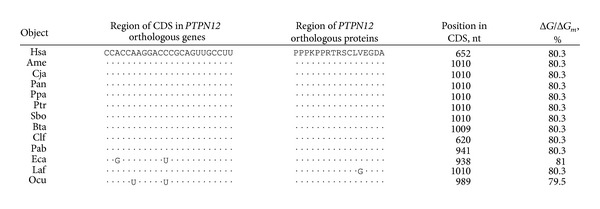

**Table 5:** Characteristics of miR-548m binding sites in CDSs of *PTPN12* orthologous genes and the variability of amino acids of PTPN12 orthologous proteins in regions with EATDI oligopeptide.

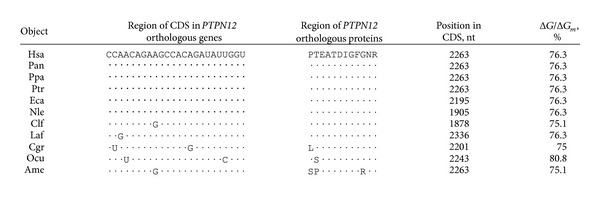

**Table 6:** Characteristics of miR-548d-5p binding sites in CDSs of *PTPN12* orthologous genes and the variability of amino acids of PTPN12 orthologous proteins in regions with STASAT oligopeptide.

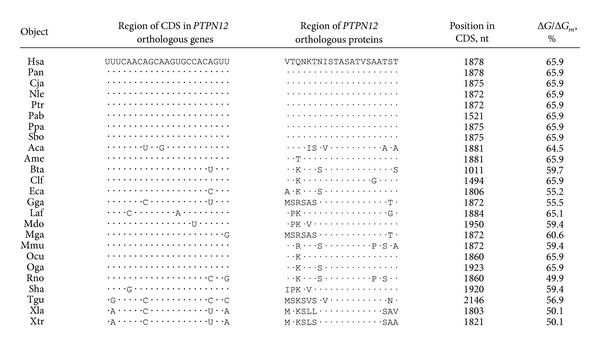

**Table 7:** Schemes of hsa-miR-1279 biding with mRNA of *PTPN12, MSH6,* and *ZEB1* orthologous genes, and schemes of hsa-miR-548j, hsa-miR-548m, and hsa-miR-548d-5p biding with *PTPN12* orthologous gene.

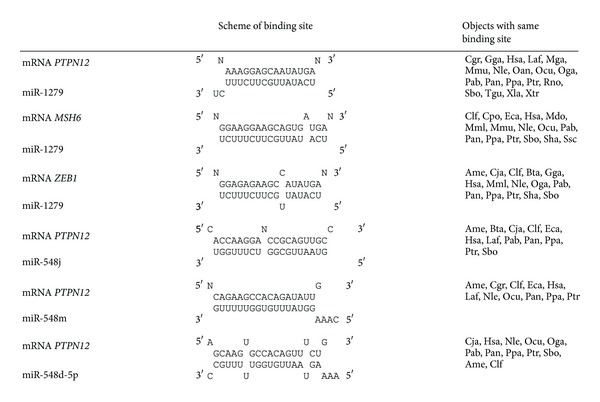

Note: N: any nucleotide—A, G, U, or C nucleotides.
