# Supplementary material for: miR-1279, miR-548j, miR-548m, and miR-548d-5p Binding Sites in CDSs of Paralogous and Orthologous PTPN12, MSH6, and ZEB1 Genes
Source: Biomed Res Int. 2013 Jul 16;2013:902467. doi: 10.1155/2013/902467 (PMC3730384; doi:10.1155/2013/902467)
Supplement: Supplementary file 1 — Accession numbers of studied orthologous genes are presented in Supplementary Table S1, S2. [file 902467.f1.docx]

**Supplementary Table S1**. Accession of *PTPN12, MSH6, ZEB1* human orthologous genes

***PTPN12*:** XM_003221488.1, Aca; XM_002919661.1, Ame; NM_001205991.1, Bta; XM_002751667.1, Cja; XM_003432299.1, Clf; NM_200669.1, Dre; XM_001915066.1, Eca; XM_415970.3, Gga; NM_001131008.1, Hsa; XM_003407183.1, Laf; NW_001115575.1, Mdo; NM_011203.2, Mga; XM_001371009.2, Mmu; XM_003268189.1, Nle; XM_001507411.2, Oan; XM_002712097.1, Ocu; XM_003782804.1, Oga; XM_003445026.1, Oni; XM_002818305.1, Pab; XM_003896407.1, Pan; XM_003805268.1, Ppa, XM_003318551.1, Ptr; NM_057115.2, Rno; XM_003921134.1, Sbo; XM_003771470.1, Sha; XR_053986.1, Tgu; NM_001091372.1, Xla; NM_001030495.1, Xtr.

***MSH6*:** XM_003216125.1, Aca; XM_002912423.1, Ame; NM_001192737.1, Bta; XM_003499545.1, Cgr; XM_003473077.1, Cpo; XM_531814.3, Clf; NM_182860.1, Dre; XM_001497961.2, Eca; NM_000179.2, Hsa; XM_003417585.1, Laf; XM_001382140.1, Mdo; XM_001113749.2, Mml; NM_010830.2, Mmu; XM_003262562.1, Nle; XM_002709880.1, Ocu; XM_002812046.2, Pab; XM_003908634.1, Pan; XM_003822683.1, Ppa; XM_003309053.1, Ptr; XM_003922758.1, Sbo; XM_003758295.1, Sha; XM_003354836.2, Ssc*.*

***ZEB1*:** XM_003216125.1, Aca; XM_002920455.1, Ame; NM_001206590.1, Bta; XM_002750141.1, Cja; XM_003433703.1, Clf; NM_131709.1, Dre; NM_205131.1, Gga; NM_001174093.1, Hsa*;* NM_001128128.2, Laf; XM_001089463.2, Mml; NM_011546.3, Mmu; XM_003276020.1, Nle; NM_001131215.2, Pab; XM_003903526.1, Pan; XM_003814801.1, Ppa; XM_003312512.1, Ptr; XM_003786729.1, Oga; NM_013164.1, Rno; XM_003938384.1, Sbo; XM_003771842.1, Sha; NM_001015808.1, Ssc; NM_001092493.1, Tgu.

**Supplementary Table S2.** Accession of *PTPN12, MSH6, ZEB1* paralogous genes

**Hsa:** *PTPN1* NM_002827.2; *PTPN2* NM_001207013.1, *PTPN4* NM_002830.2; *PTPN5* NM_006906.1; *PTPN6* NM_002831.5, *PTPN7* NM_002832.3; *PTPN9* NM_002833.2; *PTPN10* NM_004417.3; *PTPN11* NM_002834.3; *PTPN13* NM_080683.2; *PTPN14* NM_005401.4; *PTPN18* NM_014369.3; *PTPN21* NM_007039.3; *PTPN22* NM_015967.5; *MSH2* NM_000251.1; *MSH3 NM_002439.3; MSH4* NM_002440.3; *MSH5* NM_025259.5; *MLH1* NM_000249.3; *MLH3* NM_001040108.1; *ZEB2* NM_014795.3; *ZNF8* NM_021089.2; *ZNF70* NM_021916.2; *ZNF91* NM_003430.2; *ZNF148* NM_021964.2; *ZNF208* NM_007153.3; *ZNF288* NM_001164342.1; *ZNF552* NM_024762.3; *ZNF729* NM_001242680.1; *ZNF768* NM_024671.3.

**Pab:** *ZEB2* NM_001131548.1; *ZNF8* NM_001159793.1; *ZNF70* XM_002830906.1
